# Significant functional tricuspid regurgitation portends poor outcomes in patients with atrial fibrillation and preserved left ventricular ejection fraction

**DOI:** 10.1186/s12872-020-01716-6

**Published:** 2020-10-06

**Authors:** Natthaporn Prapan, Nithima Ratanasit, Khemajira Karaketklang

**Affiliations:** 1 Division of Cardiology, Department of Medicine, Faculty of Medicine, Siriraj Hospital, Mahidol University, Bangkok, 10700 Thailand; 2 Department of Medicine, Faculty of Medicine, Siriraj Hospital, Mahidol University, Bangkok, 10700 Thailand

**Keywords:** Atrial fibrillation, Functional tricuspid regurgitation, Left atrial volume, Prognosis, Pulmonary hypertension, Severe tricuspid regurgitation

## Abstract

**Background:**

Significant tricuspid regurgitation (TR) can be found in patients with atrial fibrillation (AF). The results of previous studies are controversial about whether significant functional TR (FTR) in patients with AF leads to worse clinical outcomes. The aims of the study were to investigate the prevalence, predictors and prognosis of significant FTR in patients with AF with preserved left ventricular ejection fraction (LVEF).

**Methods:**

The present study was a retrospective cohort study in patients with AF and preserved LVEF from May 2013 through January 2018. Significant FTR was defined as moderate to severe TR without structural abnormality of the tricuspid valve. Pulmonary hypertension (PH) was defined as pulmonary artery systolic pressure ≥ 50 mmHg or mean pulmonary artery pressure ≥ 25 mmHg determined by echocardiography. The adverse outcomes were defined as heart failure and death from any cause within 2 years of follow up.

**Results:**

A total of 300 patients with AF (mean age 68.8 ± 10.8 years, 50% male) were included in the study. Paroxysmal and non-paroxysmal AF were reported in 34.7 and 65.3% of patients, respectively. Mean LVEF was 65.3 ± 6.3%. PH and significant FTR were observed in 31.3 and 21.7% of patients, respectively. Patients with significant FTR were significantly older, more female gender and non-paroxysmal AF, and had higher left atrial volume index and pulmonary artery pressure than those without. A total of 26 (8.7%) patients died and heart failure occurred in 39 (13.0%) patients. There was a statistically significant difference in the adverse outcomes between patients with significant and insignificant FTR (44.6% vs. 11.9%, *p* <  0.010). Multivariable analysis showed that factors associated with significant FTR were female gender, presence of PH and left atrial volume index (OR = 2.61, 1.87, and 1.04, respectively). The predictors of the adverse outcomes in patients with AF were significant FTR, presence of PH and high CHA_2_DS_2_-VASc score (OR = 5.23, 2.23 and 1.60, respectively).

**Conclusions:**

Significant FTR was common in patients with AF, and independently associated with adverse outcomes. Thus, comprehensive echocardiographic assessment of FTR in patients with AF and preserved LVEF is fundamental in determining the optimal management.

## Background

Atrial fibrillation (AF) occurs commonly, especially with advancing age, and it is increasingly recognized as the leading cause of cardiovascular morbidity and mortality [1–3]. AF per se leads to atrial and annular dilation despite normal ventricular systolic function and size, and subsequently, secondary or functional mitral regurgitation and/or tricuspid regurgitation (TR) [4–6]. Moderate to severe functional TR (FTR) is becoming a major concern in patients with AF since it may lead to progressive right ventricular (RV) dilatation and failure, and an increase in morbidity and mortality. The prevalence of significant FTR in patients with AF varies among different studies, ranging from 5 to 35% [7–10]. A large number of previous studies have shown the association between AF and atrial functional mitral regurgitation, and its prognostic value, while only a few studies have focused on significant atrial FTR. Abe et al. showed that the prevalence of significant FTR increased with the longer duration of AF and was associated with poor prognosis [9]. In contrast, Takahashi et al. reported a favorable prognosis of severe isolated TR associated with AF [7]. Therefore, the prognostic significance of FTR in patients with AF remains unsettled. The objectives of the present study were to study the prevalence and the predictors of significant FTR in patients with AF with preserved left ventricular ejection fraction (LVEF) as well as to investigate the association of FTR with the adverse outcomes.

## Methods

### Study population

This is a retrospective cohort study of patients with AF and preserved LVEF who underwent a comprehensive echocardiographic examination at Siriraj Hospital from May 2013 through January 2018. The Siriraj Institutional Review Board, Siriraj Hospital, Mahidol University (Bangkok, Thailand) approved the study protocol. The clinical characteristics, follow-up data, electrocardiographic and echocardiographic data were collected from medical records and echocardiography laboratory database. The inclusion criteria were patients who were 18 years or older, the diagnosis of AF from either electrocardiograpm or Holter monitoring, preserved LVEF (≥ 50%) and complete follow-up data for at least 2 years. Patients with the diagnosis of heart failure at the time of echocardiography, primary TR, concomitant moderate or greater degree of primary left-sided valvular disease, prosthetic valve, permanent pacemaker, cardiomyopathy, prior cardiac device implantation or cardiac surgery, history of coronary artery disease or myocardial infarction or regional wall motion abnormality on echocardiography, chronic pulmonary disease or patients with incomplete data were excluded. AF was classified as paroxysmal and non-paroxysmal. Non-paroxysmal AF included persistent and permanent AF. CHA_2_DS_2_-VASc score for estimating risk of stroke was calculated as previously described [11]. During the follow-up, data regarding heart failure, cardiac and non-cardiac death were recorded. Heart failure was defined as heart failure visit or hospitalization. The adverse outcomes included heart failure visit or hospitalization, and death from any cause.

### Echocardiography

All echocardiographic measurements were performed according to the standard guidelines and obtained by averaging at least 5 consecutive beats. The LVEF, left ventricular (LV) dimensions, LV mass and left atrial volume (LAV) were determined according to the standard methods [12, 13]. Significant FTR was defined as moderate to severe TR without structural abnormality of tricuspid valve. The severity of FTR was determined using the combination of multiple parameters, including the assessment of vena contracta, color Doppler-derived jet area, the density and contour of continuous wave Doppler spectrum, and the hepatic vein flow Doppler pattern [14–16]. Mean pulmonary artery pressure (PAP) and pulmonary artery systolic pressure (PASP) were estimated using continuous-wave Doppler spectra of pulmonary regurgitation and TR jets, respectively [17]. Pulmonary hypertension (PH) was defined as mean PAP ≥ 25 mmHg and/or PASP ≥50 mmHg. RV systolic function was assessed by tricuspid annular plane systolic excursion (TAPSE) and peak systolic myocardial velocity of tricuspid valve annulus (S’_TV_). Impaired RV systolic function was defined as TAPSE < 16 mm and/or S’_TV_ < 10 cm/s [17].

### Statistical analysis

Descriptive statistics, including frequency and percentage, were used for categorical variables. Continuous variables were reported as mean ± standard deviation for normally distributed variables and median (percentile 25 and percentile 75) for non-normally distributed variables. Comparisons of categorical variables between patients with significant and insignificant FTR and between patients with and without adverse outcomes were performed using Chi-square test or Fisher’s exact test. Continuous variables were compared using Student’s t-test. The following variables (age, female gender, non-paroxysmal AF, dyslipidemia, diuretic, LV diastolic dimension index, LAV index, TAPSE and PASP) were analyzed to determine the factors associated with significant FTR. Differences between groups were considered to be significant for the variables with *p*-value < 0.05 and those variables were further analyzed by multivariate logistic regression (backward stepwise) to determine the independent factors associated with significant FTR and presented as odds ratio (95% confidence interval). The affection factors with the adverse outcomes and heart failure outcome were compared using the Log rank test and presented by Kaplan-Meier survival curve. Variables with *p*-value < 0.05 on the univariate analysis for adverse outcomes (eg. age, female gender, CHA_2_DS_2_-VASc score, history of stroke, history of heart failure, diuretics, LAV index, significant FTR, impaired RV systolic function and PH) and for heart failure outcome (female gender, CHA_2_DS_2_-VASc score, history of stroke, history of heart failure, diuretics, LAV index, significant FTR, impaired RV systolic function and PH) were further analyzed and included in the multivariate predictors of adverse outcomes and heart failure outcome using the Cox proportional hazard analysis (backward stepwise) and presented as hazard ratios (95% confidence interval). For all tests performed, a two-tailed *p*-value < 0.05 was considered to be statistically significant. PASW Statistic (SPSS) 18.0 (SPSS, Inc., Chicago, IL, USA) was used to perform all statistical analyses.

## Results

A total of 498 patients with AF were considered for this study, but after applying the exclusion criteria listed above, 300 patients (mean age 68.8 ± 10.8 years, 50.0% female) were enrolled in the study. Baseline characteristics, echocardiographic data and adverse outcomes in all patients are shown in Table 1.

**Table 1:** Baseline characteristics, echocardiographic data and outcomes in all patients and the comparisons between patients with significant and insignificant functional tricuspid regurgitation

Variables	All patients (***n*** = 300)	Significant FTR (***n*** = 65)	Insignificant FTR (***n*** = 235)	***P***-value
**Baseline characteristics**				
Age (years)	68.8 ± 10.8	71.3 ± 10.3	68.1 ± 10.9	0.034
Female gender	150 (50.0)	45 (68.2)	105 (44.7)	< 0.001
Non-paroxysmal AF	196 (65.3)	53 (81.5)	143 (60.9)	0.010
CHA_2_DS_2_-VASc score	3.3 ± 1.7	3.6 ± 1.8	3.2 ± 1.7	0.103
Hypertension	235 (78.3)	50 (76.9)	185 (79.1)	0.710
Dyslipidemia	153 (51.0)	24 (36.9)	129 (54.9)	0.010
Diabetes mellitus	105 (35.0)	17 (26.2)	88 (37.4)	0.091
History of stroke	88 (29.3)	18 (28.1)	70 (29.8)	0.796
History of heart failure	45 (15.0)	11 (16.9)	34 (14.5)	0.624
Diuretics	58 (19.3)	20 (30.8)	38 (16.2)	0.020
**Echocardiographic data**				
LVEF (%)	65.3 ± 6.3	63.9 ± 6.3	65.7 ± 6.3	0.052
LVDdi (mm/m^2^)	25.7 ± 4.2	26.7 ± 4.0	25.4 ± 4.2	0.025
LVSdi (mm/m^2^)	16.4 ± 3.0	17.3 ± 3.2	16.3 ± 5.0	0.137
LAV index (ml/m^2^)	49.0 ± 15.6	58.4 ± 17	46.5 ± 14.2	< 0.001
TAPSE (mm)	19.5 ± 3.1	18.7 ± 2.7	19.7 ± 3.1	0.016
S’_TV_ (cm/s)	11.4 ± 2.2	11.3 ± 2.5	12.1 ± 6.9	0.431
RAP (mmHg)	7.6 ± 3.4	9.2 ± 4.2	7.2 ± 3.0	< 0.001
Mean PAP (mmHg)	23.7 ± 7.2	26.8 ± 8.2	22.7 ± 6.5	0.002
PASP (mmHg)	38.3 ± 11.5	45.0 ± 14.7	36.3 ± 9.5	< 0.001
**Outcomes**				
Death and heart failure	57 (19.0)	29 (44.6)	28 (11.9)	< 0.001
Heart failure visit	39 (13.0)	24 (36.9)	15 (6.4)	< 0.001
Heart failure hospitalization	20 (6.7)	9 (13.8)	11 (4.7)	0.020
Death	26 (8.7)	8 (12.3)	18 (7.7)	0.240

Data are expressed as number (percentage) and mean ± standard deviation

*Abbreviations*: *AF* atrial fibrillation, *FTR* functional tricuspid regurgitation, *LAV* left atrial volume, *LVDdi* left ventricular diastolic dimension index, *LVEF* left ventricular ejection fraction, *LVSdi* left ventricular systolic dimension index, *PAP* pulmonary artery pressure, *PASP* pulmonary artery systolic pressure, *RAP* right atrial pressure, *S’*_*TV*_ peak systolic myocardial velocity of lateral tricuspid annulus, *TAPSE* tricuspid annular plane systolic excursion

### Patient characteristics

Non-paroxysmal and paroxysmal AF were reported in 65.3 and 34.7% of patients, respectively. The mean AF duration was 26.6 ± 41.3 months. The mean CHA_2_DS_2_-VASc score was 3.3 ± 1.7 (range 0–7). Anticoagulants and aspirin were prescribed in 78.3 and 12.0% of patients, respectively. Regarding concurrent medications, beta-blockers, diuretic, digoxin and amiodarone were reported in 71.3, 19.3, 6.3 and 3.7% of patients, respectively.

### Echocardiographic data

PH and significant FTR were reported in 31.3 and 21.7% of patients, respectively. About one fourth of the patients (24.3%) had no TR. The mean PAP and PASP were 23.7 ± 7.2 and 38.3 ± 11.5 mmHg, respectively. Impaired RV systolic function was reported in 14.7% of patients. TAPSE and S’_TV_ were 19.5 ± 3.1 mm and 11.4 ± 2.2 cm/s, respectively.

### Outcomes

The median follow-up duration was 2.82 years (interquartile range 1.96–4.14 years). Adverse outcomes were observed in 57 (19.0%) of patients. Heart failure occurred in 39 (13.0%) of patients, while 26 (8.7%) died. No patients suffered a cardiac death. Most deaths were from infectious cause and/or sepsis. Visits and hospitalizations for heart failure occurred in 39 (13.0%) and 20 (6.7%) patients, respectively.

### Comparisons between patients with significant and insignificant tricuspid regurgitation

Table 1 shows the comparisons between patients with significant and insignificant FTR, in term of baseline characteristics, echocardiographic data and adverse outcomes. History of stroke and heart failure were not significantly different between the two groups. The AF duration was 9.6 and 2.9 months in patients with significant and insignificant FTR, respectively (*p* = 0.078). PH and impaired RV systolic function were more prevalent in patients with significant FTR (49.2% vs. 26.2%, *p* <  0.001 and 24.6% vs. 11.9%, *p* = 0.010, respectively).

### Comparisons between patients with and without adverse outcomes

Table 2 shows the comparisons between patients with and without the adverse outcomes. Patients with adverse outcomes were older, more female gender and have higher CHA_2_DS_2_-VASc score and more comorbidities than those without. LVEF and LV dimensions were not significantly different between patients with and without adverse outcomes. There was no statistically significant difference in adverse outcomes between patients with paroxysmal and non-paroxysmal AF (14.4% vs. 21.4%, *p* <  0.141).

**Table 2:** Comparisons of baseline characteristics and echocardiographic data between patients with and without adverse outcomes

Variables	Adverse outcomes (***n*** = 57)	No adverse outcomes (***n*** = 243)	***P***-value
**Baseline characteristics**			
Age (year)	71.8 ± 10.4	68.1 ± 10.9	0.019
Female gender	42 (73.7)	108 (44.4)	< 0.001
Non-paroxysmal AF	42 (73.7)	154 (63.4)	0.192
CHA_2_DS_2_-VASc score	4.3 ± 1.9	3.0 ± 1.6	< 0.001
Hypertension	49 (86.0)	186 (76.9)	0.132
Dyslipidemia	26 (45.6)	127 (52.3)	0.366
Diabetes mellitus	25 (43.9)	80 (32.9)	0.119
History of stroke	27 (48.2)	61 (25.1)	0.001
History of heart failure	15 (26.3)	30 (12.3)	0.008
Diuretics	25 (43.9)	33 (13.6)	< 0.001
**Echocardiographic data**			
Significant FTR	29 (50.9)	36 (14.8)	< 0.001
LVEF (%)	65.6 ± 7.5	65.2 ± 6.0	0.635
LVDdi (mm/m^2^)	25.7 ± 5.9	25.7 ± 3.7	0.994
LVSdi (mm/m^2^)	16.5 ± 3.6	16.5 ± 4.9	0.876
LAV index (ml/m^2^)	54.0 ± 16.2	47.9 ± 15.3	0.008
TAPSE (mm)	18.6 ± 2.9	19.7 ± 3.1	0.027
S’_TV_ (cm/s)	13.5 ± 13.4	11.5 ± 2.2	0.073
Impaired RV systolic function	15 (26.3)	29 (11.9)	0.006
RAP (mmHg)	8.6 ± 3.9	7.4 ± 3.2	0.015
Mean PAP (mmHg)	27.2 ± 8.5	22.8 ± 6.5	< 0.001
PASP (mmHg)	42.9 ± 13.3	37.1 ± 10.8	0.001
Pulmonary hypertension	28 (49.1)	64 (26.3)	0.001

Data are expressed as number (percentage) and mean ± standard deviation

*Abbreviations*: *AF* atrial fibrillation, *FTR* functional tricuspid regurgitation, *LAV* left atrial volume, *LVDdi* left ventricular diastolic dimension index, *LVEF* left ventricular ejection fraction, *LVSdi* left ventricular systolic dimension index, *PAP* pulmonary artery pressure, *PASP* pulmonary artery systolic pressure, *RAP* right atrial pressure, *RV* right ventricular, *S’*_*TV*_ peak systolic myocardial velocity of lateral tricuspid annulus, *TAPSE* tricuspid annular plane systolic excursion

### Predictors of significant tricuspid regurgitation

Table 3 shows the univariate and multivariable predictors of significant FTR in patients with AF. Female gender, LAV index and presence of PH were independent determinants of significant FTR.

**Table 3:** Univariate and multivariable analyses of factors associated with significant functional tricuspid regurgitation

Factors	Univariate analysis			Multivariable analysis		
	OR	95%CI	***P***-value	OR	95%CI	***P***-value
Age	1.03	1.00–1.06	0.035			
Female gender	2.78	1.56–5.00	0.001	2.61	1.40–4.88	0.003
Non-paroxysmal AF	2.84	1.44–5.60	0.003			
LAV index	1.05	1.03–1.07	< 0.001	1.04	1.01–1.06	< 0.010
Impaired RV systolic function	2.41	2.21–4.80	0.010			
Pulmonary hypertension	2.73	1.5–4.82	0.001	1.87	1.01–3.46	0.046

*Abbreviations*: *AF* atrial fibrillation, *CI* confidence interval, *LAV* left atrial volume, *OR* odd ratio, *RV* right ventricular

### Predictors of adverse outcomes

**Fig. 1:**
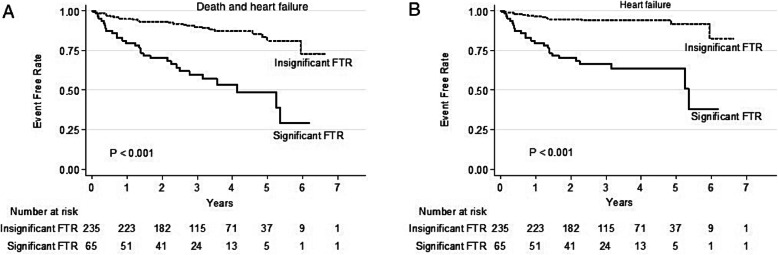
Kaplan-Meier curves for the event-free rates from death and heart failure (**a**) and heart failure (**b**) in patients with atrial fibrillation, comparisons between those with significant and insignificant functional tricuspid regurgitation. FTR = functional tricuspid regurgitation

Table 4 shows the univariate and multivariable predictors of the adverse outcomes in patients with AF. Significant FTR was the strongest predictor of adverse outcomes. Female gender, presence of PH and significant FTR were independent determinants of heart failure when that was considered as the only adverse cardiovascular outcome (Table 5). On the survival analysis, patients with significant FTR significantly had poorer prognosis that those with insignificant FTR (Fig. 1a and b). The event-free rates from death and heart failure at 1, 3, 5, and 7 years were 91.7, 83.3, 76.0 and 65.1%, respectively. The event-free rates from heart failure at 1, 3, 5, and 7 years were 92.9, 87.8, 85.7 and 76.3%, respectively.

**Table 4:** Univariate and multivariable analyses of the predictors of death and heart failure

Factors	Univariate analysis			Multivariable analysis		
	HR	95%CI	***P***-value	HR	95%CI	***P***-value
Age	1.03	1.01–1.06	0.013			
Female gender	3.53	1.94–6.42	< 0.001			
CHA_2_DS_2_-VASc score	1.50	1.29–1.75	< 0.001	1.47	1.25–1.73	< 0.001
History of stroke	2.49	1.47–4.19	0.001			
History of heart failure	2.04	1.13–3.68	0.019			
Diuretics	3.56	2.11–6.02	< 0.001	2.29	1.34–3.92	0.003
LAV index	1.02	1.01–1.03	0.005			
Significant FTR	4.78	2.84–8.05	< 0.001	3.62	2.11–6.21	< 0.001
Impaired RV systolic function	2.20	1.22–3.98	0.009			
Pulmonary hypertension	2.68	1.59–4.51	< 0.001	2.10	1.22–3.60	0.007

*Abbreviations*: *CI* confidence interval, *FTR* functional tricuspid regurgitation, *LAV* left atrial volume, *RV* right ventricular, *HR* Hazard ratio

**Table 5:** Univariate and multivariable analyses of the predictors of heart failure visit and hospitalization

Parameters	Univariate analysis			Multivariable analysis		
	HR	95%CI	***P***-value	HR	95%CI	***P***-value
Female gender	4.04	1.89–8.63	< 0.001	2.41	1.12–5.22	0.025
CHA_2_DS_2_-VASc score	1.32	1.10–1.58	0.003			
History of stroke	2.02	1.07–3.82	0.030	2.05	1.06–4.0	0.034
History of heart failure	2.87	1.47–5.61	0.002			
Diuretics	5.86	3.11–11.07	< 0.001	3.32	1.71–6.46	< 0.001
LAV index	1.03	1.01–1.04	< 0.001			
Significant FTR	7.08	3.70–13.54	< 0.001	4.59	2.33–9.05	< 0.001
Impaired RV systolic function	2.75	1.39–5.44	0.004			
Pulmonary hypertension	3.87	2.04–7.37	< 0.001	2.44	1.25–4.79	0.009

*Abbreviations*: *AF* atrial fibrillation, *CI* confidence interval, *FTR* functional tricuspid regurgitation, *LAV* left atrial volume, *RV* right ventricular, *HR* Hazard ratio

## Discussion

The present study demonstrates that moderate to severe FTR portends poor prognosis in patients with AF and preserved LVEF, especially in terms of heart failure. Female gender, LAV index and presence of PH were independent predictors of significant FTR. Furthermore, CHA_2_DS_2_-VASc score, significant FTR and presence of PH were independent predictors of heart failure and death. Interestingly, the presence of PH was the only parameter in common to predict both significant FTR and adverse outcomes.

### Prevalence of significant functional tricuspid regurgitation in patients with atrial fibrillation

The prevalence of significant FTR in the present study was 21.7%. The reported prevalence of FTR in patients with AF has varied among different studies and depends on several factors, including the size and demographics of the study population, AF duration, the measurement and definition of significant FTR, different echocardiographic techniques (two-dimensional vs. three-dimensional, or transthoracic vs. transesophageal echocardiography) [7–9, 18–20]. Abe et al. demonstrated the relationship between AF duration and the prevalence of significant FTR (0, 17 and 25% of patients at < 1, 1–10 and > 10 years, respectively) [9]. The present study demonstrated that non-paroxysmal AF was more common in patients with significant FTR than those with insignificant FTR, which supports the possible explanation that long-standing AF may lead to right atrial dilatation and eventually, significant atrial FTR.

### Importance of significant tricuspid regurgitation and pulmonary hypertension in patients with atrial fibrillation

The presence of significant FTR is associated with an increase in morbidity and mortality, regardless of pulmonary pressure and LVEF [21]. FTR is not uncommon and occurs as a result of various underlying diseases, such as left-sided heart disease or PH [18, 19, 22]. Recently, isolated FTR in patients with AF has gained much attention. However, some concerns have arisen regarding the long-term prognostic significance and the optimal management of atrial FTR. Some previous studies reported the favorable short-term prognosis of FTR in patients with AF. For example, Yakahashi et al. demonstrated a good prognosis of idiopathic FTR in patients with AF without structural heart disease and no PH with the 1-year cumulative mortality and cumulative mortality due to right-sided heart failure of 5 and 1%, respectively [7]. The present study shows that the cumulative adverse outcomes after 2 years are higher. In this population, 44.6% of the patients had adverse outcomes, including heart failure (36.9%) and death (12.3%). Of note, the presence of PH worsens the prognosis of patients with significant TR [21].

There was no cardiac death over the two-year duration of the present study or the one-year duration of the previous study by Yakahashi et al. [7]. Apart from the short-term follow-up in these studies, the explanation may be, in part, due to a benign nature of FTR (unless associated with PH, RV or LV dysfunction) and the long duration of optimal treatment with medication. Even though there was no cardiac death, the present study found that heart failure was an important cardiovascular endpoint. Our findings demonstrated that heart failure occurred in 36.9% of AF patients with significant FTR over the course of 2 years. Previous studies have reported a poor prognosis in patients who have AF with heart failure, even with preserved LVEF [1]. This is true as well for patients with AF and persistent TR who required hospitalization for right-sided heart failure [7].

### Predictors of significant functional tricuspid regurgitation in patients with atrial fibrillation

FTR has been described in a variety of cardiac disorders with RV remodeling and tricuspid annular dilatation [19, 23, 24]. AF leads to atrial enlargement and dilatation of mitral and tricuspid annuli [4]. Advanced age and right heart enlargement have been associated with atrial FTR [8, 18, 20]. Furthermore, some echocardiographic parameters are related to TR severity in patients with AF, such as LAV index, tricuspid annular diameter, tenting height, right atrial area, PASP and RV remodeling [8, 25]. Apart from the echocardiographic parameters related to right heart remodeling, the present study demonstrated that female gender, LAV index and PH were independently associated with significant FTR in patients with AF. Left atrial enlargement and an increase in PAP reflect the non-right heart disease, which indirectly leads to FTR. Diastolic dysfunction is a common functional cardiac abnormality leading to left atrial enlargement and PH, and possibly FTR. However, further studies will be required to elucidate this causal relationship.

### Predictors of adverse outcomes in patients with atrial fibrillation

The prognostic significance of significant FTR in patients with AF was previously described, and an increasing severity of TR was correlated with a worse outcome [21, 26]. Data from a community-based study showed that AF in patients with heart failure and preserved LVEF portended a poor prognosis [1]. Significant FTR increased the probability of future episodes of heart failure in patients with AF [9]. In agreement with other studies, the present study showed that significant FTR was an independent predictor of adverse outcomes of heart failure and death. Furthermore, patients with significant FTR were more commonly found to have worse hemodynamic parameters, such as right atrial pressure, PAP, the presence of PH and impaired RV systolic function. These findings might help to explain the more adverse outcomes found in this group. Data from the present study suggest that significant FTR merits further study in an attempt to determine ways to decrease the incidence of significant FTR and thus to prevent the high incidence of adverse outcomes reported in the present study.

### Study limitations

This was a retrospective study conducted in a single center. A larger, prospective, multicenter study of patients with AF should be encouraged to identify the accurate incidence of FTR and factors associated with adverse outcomes. Most of the measurements in this study were performed using two-dimensional echocardiography, which might be less accurate and less reproducible than three-dimensional echocardiography. Specifically, TR severity was routinely assessed using the vena contracta width, TR jet area or semiquantitatively by visual estimation. More accurate assessment of TR severity, such as proximal isovelocity surface area, should be performed. Due to the retrospective nature of the present study, the echocardiographic findings did not include the parameters of right heart remodeling and could not elucidate the causal relationship between AF and atrial FTR.

## Conclusions

Significant FTR was common in patients with AF and associated with adverse outcomes. Female gender, LAV index and presence of PH were independent predictors of significant FTR, while high CHA_2_DS_2_-VASc score, significant FTR and presence of PH were independent predictors of the adverse outcomes in patients with AF and preserved LVEF.

## Data Availability

The datasets used and/or analysed during the current study are available from the corresponding author on reasonable request.

## Abbreviations

AF: Atrial fibrillation
FTR: Functional tricuspid regurgitation
LAV: Left atrial volume
LV: Left ventricular
LVEF: Left ventricular ejection fraction
PAP: Pulmonary artery pressure
PASP: Pulmonary artery systolic pressure
PH: Pulmonary hypertension
RV: Right ventricular;
S’_TV_: Peak systolic myocardial velocity of lateral tricuspid annulus
TAPSE: Tricuspid annular plane systolic excursion
TR: Tricuspid regurgitation
